# Development of Value-Added Food Products from European Catfish (*Silurus glanis*) to Support Its Valorisation and Capture in the Tagus River, Portugal

**DOI:** 10.3390/foods15152667

**Published:** 2026-07-29

**Authors:** João Reis, Irene Cristina Antunes, Gabriela Basto de Lima, Ana Teresa Ribeiro, Ana Neves, Luísa Cristina Roseiro, Nuno Alvarenga, Helena Gonçalves, Jaime Fernandes, Mafalda Silva, Elsa M. Gonçalves, Sandra Fernandes Gomes, Manuela Lageiro, Andreia Soares, Paula Pinto, Igor Dias

**Affiliations:** 1 School of Agriculture, Santarém Polytechnic University, Quinta do Galinheiro—S. Pedro, 2001-904 Santarém, Portugal; maria.lima@esa.ipsantarem.pt (G.B.d.L.); igor.dias@esa.ipsantarem.pt (I.D.); 2 UEISTSA—Unidade Estratégica de Investigação e Serviços de Tecnologia e Segurança Alimentar, Instituto Nacional de Investigação Agrária e Veterinária (INIAV), 2780-157 Oeiras, Portugalcristina.roseiro@iniav.pt (L.C.R.); nuno.alvarenga@iniav.pt (N.A.); helena.goncalves@iniav.pt (H.G.);; 3 CIEQV—Life Quality Research Centre, Avenida Dr. Mário Soares n 110, 2040-413 Rio Maior, Portugal; 4 GeoBioTec—Geobiociências, Geoengenharias e Geotecnologias, FCT-UNL, 2829-516 Caparica, Portugal; 5 Faculdade de Ciências e Tecnologia, Universidade Nova de Lisboa, Campus da Caparica, 2829-516 Caparica, Portugal; 6 Research Center in Natural Resources, Environment and Society (CERNAS), Santarém Polytechnic University, Quinta do Galinheiro—S. Pedro, 2001-904 Santarém, Portugal

**Keywords:** new products, nutritional analyses, *Silurus glanis*, sustainable protein, valorisation

## Abstract

The European catfish (*Silurus glanis*) is an invasive predatory species threatening biodiversity in the Tagus River (Portugal), while remaining underexploited as a food resource. Despite its potential contribution to invasive species management, limited knowledge exists on its suitability for developing value-added food products, particularly using processing scraps. This study aimed to valorise this species through the development and characterisation of six value-added food products produced from fillets and processing scraps. Nutritional, microbiological, textural, and sensory properties were evaluated. Microbiological analyses confirmed the absence of *Salmonella* spp., *Listeria monocytogenes*, and *E. coli* in all products; however, the high microbial counts observed in Powerbites indicate the need for improved hygiene and process control. Protein content ranged from 10.05 to 16.89 g/100 g, with complete essential amino acid profiles and relevant contributions to adult daily indispensable amino acid requirements. The products also showed low to moderate fat content, favourable fatty acid profiles, and meaningful contributions of vitamins B12 and E. Sensory evaluation indicated overall positive acceptance, although some formulations require further optimisation. These findings highlight the nutritional, technological, and sustainable potential of European catfish-based products, supporting their use as value-added food alternatives while contributing to the valorisation of an underused invasive species.

## 1. Introduction

*Silurus glanis*, commonly known as the European catfish, is one of the largest freshwater fish species in Europe, reaching up to 3 m in length and capable of exceeding 300 kg in weight [1]. This predatory fish is native to the slow-moving rivers and lakes of Eastern Europe and parts of Asia, where it thrives in murky waters. It is an opportunistic feeder, primarily preying on fish, invertebrates, and small mammals [2]. *Silurus glanis* is believed to have spread across Europe mainly due to intentional introductions for sport fishing and aquaculture purposes, but also as inadvertent translocations [3]. The fish’s adaptability and voracious feeding habits have allowed it to establish populations in various countries, including those where it was artificially introduced, potentially disrupting local ecosystems. Portugal is one of the countries invaded by this species of catfish. It has become an invasive species threatening the native aquatic ecosystem [4]. The fish’s presence in Portuguese waters disrupts local biodiversity, preying on native fish species and competing for resources. Its rapid spread is of particular concern, as it lacks natural predators and its population grows unchecked. In Portugal, the fish has not yet been fully integrated into the local diet, largely due to the absence of a traditional consumption culture for this species. Consequently, its impact on the ecosystem is a consequence of the fact that local communities do not actively fish for the species.

Despite its invasive nature, *Silurus glanis* holds potential as a food source with both nutritional and gastronomic value. Its white, firm flesh is mild in flavour and can be compared to other freshwater fish such as pike or bass [5]. From a nutritional perspective, *S. glanis* may represent a relevant freshwater fish resource, providing nutrients of interest for human consumption, particularly protein and lipids with potential nutritional value. These general characteristics support its consideration as an alternative raw material and justify further investigation into its application in value-added food products. With proper promotion, its culinary qualities could contribute to market uptake and increased consumption, which may, in turn, encourage targeted fishing efforts to control its population and mitigate its ecological impact [6]. In this context, the food valorisation of invasive species has recently been proposed as a complementary strategy for sustainable management, particularly when harvesting is combined with product development, consumer acceptance, and circular use of biomass. Such approaches may help transform ecological pressure into nutritional and economic opportunities, while supporting more sustainable food innovation [7,8,9].

Previous studies on fish valorisation have shown that low-commercial-value and/or unexploited species can be used to develop value-added seafood products. In Portugal, Silva et al. [10] developed new products using low-commercial-value and unexploited marine species from the Portuguese coast, while Duarte et al. [11] assessed the nutritional composition of unexploited and low-commercial-value fish species. Nevertheless, these studies did not consider invasive freshwater species or the combined use of fillets and edible processing by-products for food product development.

The processing of *Silurus glanis* follows the typical procedures for large fish, including washing, filleting, and gutting. The washing step is especially important, since this type of fish does not possess scales and, as such, produces larger quantities of mucus to defend itself [12,13]. Said mucus must be thoroughly washed to prevent contamination and flavour alteration. The fish’s meat is highly valued for its texture and versatility in various culinary preparations, such as fillets and smoked fish [5]. However, a significant portion of the fish is discarded, including the bones, skin, and some meat scraps. These portions, traditionally viewed as waste, have untapped potential for further use. A part of this waste, like skin and bones, can be processed into value-added products, such as fishmeal, gelatin, or collagen, and offer sustainable alternatives for industries like animal feed, cosmetics, and pharmaceuticals [14]. Although fish scraps lack the appeal of fish fillets, they still present all the nutritional benefits. The development of compound products would further value the consumption of *Silurus glanis* and open the way for more diverse recipes. The development of innovative products from these could enhance the economic value of the fish, making their fishing more profitable. Additionally, promoting the utilisation of fish by-products would not only help reduce waste but also contribute to sustainable practices in the fishing industry.

The increase in awareness and consumption of *Silurus glanis* could encourage its fishing [7]. This encouragement, in Portugal and other regions affected by this invader, can help mitigate its ecological impact [6,14]. By recognising its potential as a food source and utilising fish by-products, this invasive species can be transformed into an economic asset. This approach would promote the sustainable management of fish populations, aiding in the control of this invasive predator and helping restore native ecosystems while reducing the pressure on local fish stocks and boosting local economies.

This study aimed to develop and comprehensively characterise value-added food products derived from *Silurus glanis*. The developed products were evaluated in terms of nutritional composition, physical properties, microbiological safety, and sensory acceptance. To the best of our knowledge, this represents the first study conducted in Portugal addressing the valorisation of this invasive species through the development and assessment of food products obtained from fillets and accrued edible fish scraps.

## 2. Materials and Methods

### 2.1. Sample Processing and Product Development

**Figure 1:**
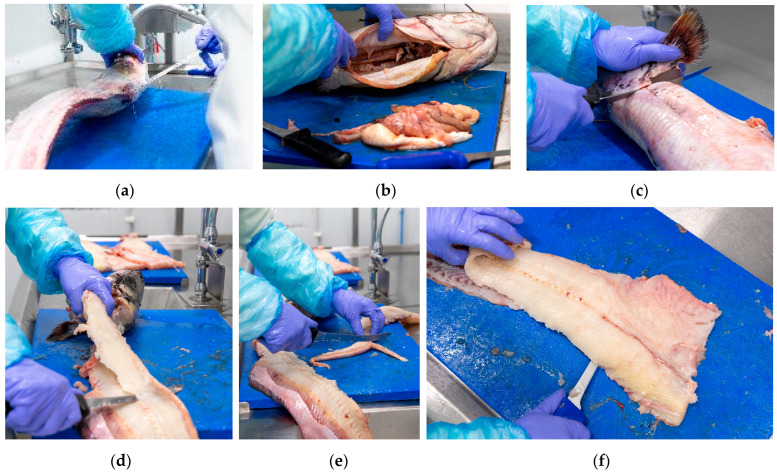
Processing workflow used for the preparation of *S. glanis*. The main steps included (**a**) Gutting; (**b**) Cleaning the gutted area; (**c**) Cutting; (**d**) Retrieval of fish flesh scraps for not having the desired dimensions; (**e**) Trimming unwanted fat from edible flesh scraps; (**f**) Filleting.

*S. glanis* specimens were captured in the Belver Reservoir (Tagus River, Portugal) and transported to Vivid Foods (Atalaia, Portugal) on the same day as capture. Transportation was carried out in an insulated vehicle, with the fish fully covered with flake ice to maintain the flesh temperature below 4 °C. The distance between the Belver Reservoir (Tagus River, Portugal) and the processing facility was approximately 49 km, corresponding to a transport time of approximately 35 min. Upon arrival, the fish were processed. A total of fifteen *Silurus glanis* specimens were used in this study, with an average live body weight of 4.38 ± 2.36 kg (range from 2.2 to 8.31 kg) and an average total length of 83.95 ± 14.92 cm (range from 66 to 112 cm). After evisceration, the specimens had a mean eviscerated body weight of 3.61 ± 1.99 kg (range from 2.10 to 7.76 kg). The fish were manually washed, gutted and filleted (Figure 1). The resulting fillets were used as a whole, and the scraps (broken fillets and/or flesh scraped from the bones) were collected and used to develop compound foods, in the form of hamburgers and “fish balls”.

Six value-added products were developed using *Silurus glanis* edible material as the main raw material. The selection of ingredients and determination of their respective proportions were carried out by the industrial partner VividFoods (Atalaia, Portugal), which was responsible for product formulation within the framework of the project. The formulations were developed based on the company’s technical expertise in food product development and the specific technological, nutritional, and sensory objectives established for each product category.

The formulations were designed to assess different product formats and technological strategies for the valorisation of both catfish fillets and processing scraps, including seasoned fillets, breaded fillets, restructured hamburgers, breaded hamburgers, and fish balls. The main technological objective was to maximise the incorporation of catfish raw material while ensuring adequate product structure, cohesion, flavour, handling properties, and suitability for packaging.

The final ingredient compositions, presented in Table 1, were subsequently used for the nutritional, technological, microbiological, and sensory characterisation of the developed products.

The selection and proportion of ingredients were defined according to their technological, nutritional, and sensory functions in fish-based products. *S. glanis* was used as the main protein source, while structuring and water-binding ingredients were included to improve cohesion, texture, and handling properties, particularly in restructured products [15,16,17]. Oils, marinades, herbs, spices, vegetables, and breading ingredients were incorporated to enhance juiciness, flavour, consumer appeal, and product differentiation [18,19]. The ingredient composition of each formulation, together with the main technological function of each ingredient group, is presented in Table 1.

**Figure 2:**
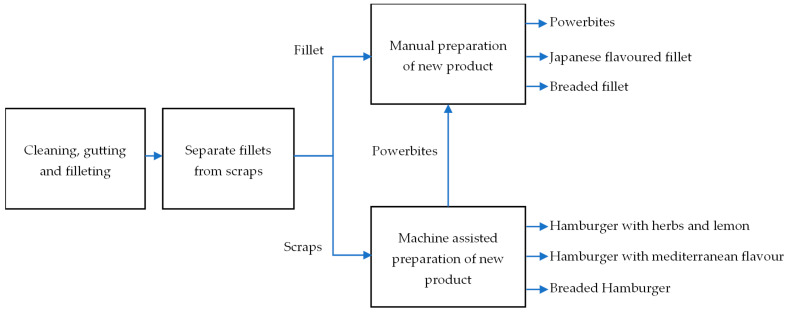
Processing workflow used for the development of *S. glanis*-based products.

**Figure 3:**
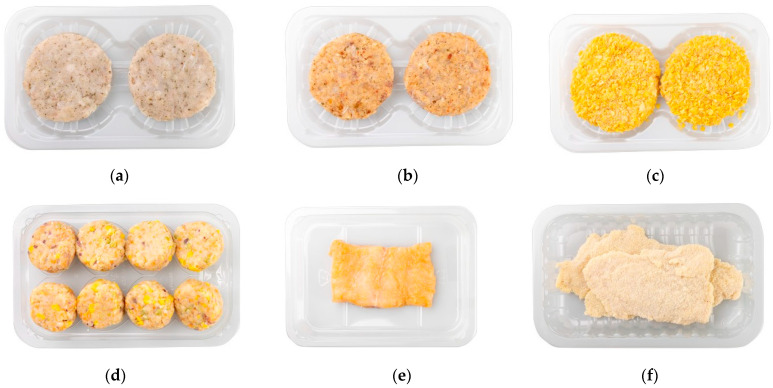
Developed products using *S. glanis*: (**a**) Hamburger with herbs and lemon; (**b**) Hamburger with Mediterranean flavour; (**c**) Breaded Hamburger; (**d**) Powerbites; (**e**) Japanese Flavoured Fillet; (**f**) Breaded Fillet.

The developed fish products were grouped according to the type of raw material and processing strategy. Whole fillets were used for the Japanese flavoured fillet (JFF) and breaded fillet (BF), whereas edible processing scraps, including broken fillets and flesh recovered from bones, were used for the restructured products: hamburger with herbs and lemon (HHL), hamburger with Mediterranean flavour (HMF), breaded hamburger (HBC), and fish balls (powerbites-PB). The hamburgers and powerbites were prepared using a horizontal bowl cutter mixer (HCH14N, GGM Gastro, Ochtrup, Germany) to homogenise the fish material with the remaining ingredients. Hamburgers were shaped and pressed using a burger-forming machine (Formatic R2200, Deighton Manufacturing Limited, Bradford, UK), whereas powerbites were manually shaped into fish balls. Fillet-based products were manually prepared, seasoned or coated according to the respective formulation. The general preparation process of the developed products can be seen in Figure 2. All products were packed in rPET containers and sealed with PET film. The final packaged products are shown in Figure 3.

### 2.2. Experimental Design

In this study, the experimental unit was the developed product, with three independent batches prepared for each product type (*n* = 3). Samples from each product were collected for nutritional, instrumental, microbiological, and sensory analyses. Chemical and instrumental measurements were carried out in triplicate for each sample, and replicate values were averaged at the batch level before statistical analysis. Thus, comparisons among products were performed using independent batch-level values. The number of independent samples included in each analysis is specified in the corresponding tables. The results are expressed as mean ± standard deviation.

### 2.3. Proximate Analysis and Energy Content

Proximate composition was determined according to AOAC 2000 [20], namely, 950.46 for moisture content, 976.06 for crude protein, 2003.05 for crude fat, 962.09 for total fibre, and 930.23 for ash. Carbohydrates were calculated by difference. Energy content was calculated from each component’s contribution using the conversion factors outlined in Annex XIV of Regulation (EU) No. 1169/2011 [21].

### 2.4. Amino Acid Profile

The amino acid composition was analysed according to the protocol previously described [22]. Briefly, 0.5 g of the sample was hydrolysed with 10 mL of 6 M HCl in sealed Pyrex tubes at 110 °C for 20 h in a vacuum oven. After cooling, the hydrolysate was filtered (Filter-Lab 110 mm) and diluted to 100 mL with distilled water. For tryptophan, 0.1 g of sample was hydrolysed with 10 mL of 4 M NaOH at 110 °C for 20 h, filtered, diluted to 100 mL, and neutralised to pH 7 with HCl. For amino acid derivatisation, 200 µL of the diluted hydrolysate was mixed with 800 µL of o-phthalaldehyde (OPA) solution and injected into an Alliance 2695 HPLC system with a 2475 fluorescence detector (Waters, Milford, CT, USA). Separation was carried out on a Spherisorb ODS2 C18 column (4.6 × 250 mm, 5 µm) at room temperature using a gradient of solvent A (0.1 M sodium acetate–methanol–tetrahydrofuran, 905:90:5) and solvent B (methanol) at 1 mL/min. Amino acids were detected by fluorescence (excitation 340 nm, emission 455 nm) and quantified by calibration curves.

Each amino acid’s contribution to meeting the amino acid consumption requirements for an average adult was calculated as proposed by a report of an FAO Expert Consultation [23]. The calculations assumed an individual of 18 years or older, weighing 70 kg, with a consumption of 100 g of fish products.

### 2.5. Vitamin Profile

The quantification of B-complex vitamins was determined according to the protocol previously described [24]. Five grams of sample were extracted with 30 mL of 0.4 M sodium acetate buffer (pH 7.4) using a Polytron homogeniser (10,000 rpm, 1 min), heated in a boiling water bath for 30 min, cooled, treated with 0.5 g taka-diastase, and incubated at 45 °C for 18 h. After the addition of 2 mL trichloroacetic acid and reheating for 5 min, the extracts were filtered and passed through a 0.45 μm hydrophilic membrane (Acrodisc, Waters, Milford, MA, USA) before HPLC analysis. The identification and quantification of B-complex vitamins (thiamine, riboflavin, niacin, pyridoxine, folic acid, and cobalamin) were achieved by Alliance 2695 separation module (Waters, Milford, MA, USA) equipped with a photodiode array detector (PDA 996, Waters, Milford, MA, USA), using a Waters Atlantis T3 C18 column (250 × 3.9 mm) in reversed phase at room temperature, and a gradient of 0.025% trifluoroacetic acid (A) and acetonitrile (B). Detection wavelengths were 245 nm (B1), 360 nm (B2), 260 nm (B3), 210 nm (B5), 290 nm (B6), 280 nm (B9), and 210 nm (B12). Quantification was performed by external calibration from peak height–concentration curves.

The tocopherol profile was analysed as previously described [25]. 5 g of sample were saponified at 80 °C for 15 min with 20 mL of saponification solution (11% *w*/*v* KOH in 55% *v*/*v* absolute ethanol and 45% *v*/*v* distilled water) and 0.6 g ascorbic acid. After cooling for 1 min, 6 mL distilled water and 12 mL n-hexane containing BHT (25 µg/mL) were added, vortexed for 2 min, and centrifuged (1500 *g*, 5 min). The upper phase was passed through anhydrous sodium sulphate and a 0.45-µm hydrophobic Acrodisc (Filter Lab, Barcelona, Spain) and injected into the HPLC system (Alliance 2695 separation module; Waters, Milford, MA, USA). Tocopherols were analysed by normal-phase HPLC using a silica column (Waters Silic, 125 × 4.6 mm, 5 μm; Waters, Milford, MA, USA) and 8% dioxane in n-hexane (*v*/*v*) as mobile phase at 1.5 mL/min. Detection was performed by fluorescence (Waters 474, Milford, MA, USA; excitation 295 nm, emission 325 nm). The injection volume was 100 μL. Tocopherols were identified by comparison of retention times with standards and quantified by external calibration.

The adequate intake (AdInt) of vitamin E and B-complex vitamins was compared to the values established by the panel on Nutrition, Novel Foods and Food Allergens of the European Food Safety Authority (EFSA NDA) for adult males ≥ 18 years (Table 2), considering a consumption of 100 g of product.

Whenever a vitamin-specific AdInt value was not stipulated, the average requirement was used instead. The contribution of 100 g of each fish product to the AdInt of a given vitamin was calculated according to the following formula:

$$\mathrm{Adequate}\;\mathrm{intake}\;(\mathrm{AdInt})=\frac{\mathrm{Fish}\;\mathrm{product}\;\mathrm{vitamin}\;\mathrm{quantity}}{\mathrm{Specific}\;\mathrm{AdInt}\;\mathrm{value}}\times 100\%$$

### 2.6. Fatty Acids Profile

Fatty acid (FA) composition was analysed following the ISO 5508:1990 [33], using gas chromatography of fatty acid methyl esters (FAME). The fat samples underwent transesterification to convert triglyceride fatty acids into FAME, following the ISO 5509:2000 [34]. Analyses were performed using a gas chromatograph (Trace GC 2000, ThermoQuest, Milan, Italy), equipped with a split injector and a flame ionisation detector, utilising a DB-23 capillary column (60 m × 0.25 mm × 0.25 µm) (J&W Scientific, Agilent Technologies, St. Clara, CA, USA). FA identification was conducted by comparing retention times with FAME reference standards. For quantitative analysis, the concentration of each FA was expressed as a percentage, calculated as the ratio of the area of the peak corresponding to the FA of interest to the total area of all peaks in the chromatogram.

#### Fatty Acid Ratios and Lipid Quality Indices

For the calculation, non-detected fatty acids were considered as zero. The FA ratios and lipid quality indices of *S. glanis* developed products were calculated as follows:

• Polyunsaturated/saturated ratio (P/S) [35]:

P/S = [(C18:2 n-6 + C18:3 n-3)/(C14:0 + C16:0 + C18:0)]

• n-3/n-6 ratio [35]:

n-6/n-3 = (∑n-6 PUFA)/(∑n-3 PUFA)

• Atherogenicity index (AI) [36]:

AI = (C12:0 + 4 × C14:0 + C16:0)/[(∑MUFA + ∑(n-6) + ∑(n-3)]

• Thrombogenicity index (TI) [36]:

TI = (C14:0 + C16:0 + C18:0)/[(0.5 × ∑MUFA + 0.5 × (n-6) + 3 × (n-3) + (n-3)/(n-6)]

• Peroxidability index (PI) [37]:

PI = (% monoenoic × 0.025) + (% dienoic × 1) + (% trienoic × 2) + (% tetraenoic × 4) + (% pentaenoic × 6) + (% hexaenoic × 8)

• Hypocholesterolemic/hypercholesterolemic ratio (h/H) [38]:

h/H = [(C18:1 n-9 + C18:2 n-6 + C18:3 n-3 + C20:4 n-6 + C20:5 n-3 + C22:5 n-3 + C22:6 n-3)/(C14:0 + C16:0)]

### 2.7. Microbiological Analysis

This study aimed to evaluate the hygienic quality and safety of the developed fish products within 12 h after their preparation, ensuring the samples’ temperature remains between 0 °C and 5 °C during transit to the laboratory. Microbiological analysis follows international standards and established procedures. Total microorganism count at 30 °C followed ISO 4833-1:2013 [39], and total microorganism count at 7 °C followed the same ISO with the modification of using a temperature of 7 °C instead of 30 °C, for 10 days to allow psychrotrophic enumeration. *Pseudomonas* spp., Enterobacteriaceae and *Escherichia coli* counts followed, respectively, ISO 13720:2022 [40], ISO 21528-2:2017 [41] and ISO 16649-2:2001 [42]. All results were expressed as colony-forming units (CFU/g). Detection analyses for *Salmonella* spp. and *Listeria monocytogenes* followed ISO 6579-1:2007 [43] and 11290-1:2017 [44], respectively, and were expressed as absent (A) or present (P).

### 2.8. Texture Analysis

Texture parameters, toughness and firmness, were determined for the raw developed products using a texture analyser (TA.XTplusC, Stable Micro Systems, Godalming, UK) with a maximum measuring force of 50 kg (500 N) and a P/50 probe (Dia Cylinder Aluminium). The used settings were: pre-test and test speed: 1.5 mm/s; post-test speed: 10.0 mm/s; distance to contact sample: 20.0 mm; trigger force: 0.1 N. The tests were set using the Exponent Connect software (Stable Micro Systems, Godalming, UK).

### 2.9. Sensory Analysis

The sensory analysis was conducted with a panel of 49 untrained adult consumers (60% women and 40% men, with ages ranging from 18 to 70 years).

It was carried out in a sensory evaluation room in accordance with the criteria outlined in ISO 8589:2007 [45]. The evaluations took place in standardised individual booths under controlled lighting conditions, with room temperature maintained at 21 ± 2 °C. Before the evaluation, all participants were informed about the nature of the samples, the evaluation procedure, and the exclusive use of the collected data for research purposes. Informed consent was obtained from all assessors before participation, and anonymity and data protection were ensured in compliance with the General Data Protection Regulation (GDPR). The developed products were prepared under standardised conditions and served in individual portions coded with random three-digit numbers to prevent identification bias. The order of sample presentation was randomised among assessors to minimise order and carry-over effects. Water was provided for palate cleansing between samples. Sensory attributes, namely appearance, colour, aroma, texture, taste, juiciness, and overall acceptance, were evaluated using a 5-point hedonic scale, where 1 corresponded to “dislike very much” and 5 to “like very much”. The use of a 5-point hedonic scale was a deliberate methodological choice, as the primary objective of the sensory evaluation was to assess overall consumer acceptability and market-oriented perception of newly developed products rather than to detect subtle sensory differences between closely related formulations. Given the exploratory nature of the study and the use of an untrained consumer panel with a wide age range, the reduced scale facilitated comprehension, minimised cognitive fatigue, and improved response consistency across assessors. Shorter hedonic scales are commonly applied in product screening and preliminary acceptability studies, particularly when products differ markedly in formulation, texture, and flavour profile [46,47,48].

In addition to sensory liking, purchase intention was evaluated using a hypothetical approach. Assessors were asked the direct question: “Would you buy this product if it were available on the market?”, and responses were recorded on a 5-point scale ranging from “certainly would not buy” (1) to “certainly would buy” (5). No price information was provided, as the aim of this question was to assess the intrinsic predisposition to purchase based solely on sensory perception, independent of economic considerations. This approach is widely used in early-stage product development to provide an indication of potential consumer acceptance rather than to predict actual market behaviour.

The sensory evaluation was conceived as an exploratory consumer screening study. Although larger and demographically stratified consumer samples are recommended for definitive acceptance testing, the number of assessors included was considered adequate to identify major differences in consumer perception among products with distinct formulations. For exploratory acceptability and product screening studies, the use of untrained consumer panels with sample sizes below 100 participants has been reported as adequate to identify major differences in consumer perception [49,50].

The sensory evaluation was conducted in three independent sessions, with the 49 consumers distributed across sessions. The same sample preparation, coding, serving temperature, and evaluation protocol were applied in all sessions.

### 2.10. Statistical Analysis

Statistical analyses were performed only for variables with sufficient quantifiable values across products. Missing values resulting from analytical or technical issues were excluded from the corresponding statistical comparison, and no imputation was performed. Data were analysed using the Statistica™ v.8.0 software from Statsoft (StatSoft Inc., 1984–2007, Tulsa, OK, USA). The assumptions of normality and homogeneity of variance were assessed using the Shapiro–Wilk and Levene’s tests, respectively. One-way analysis of variance (ANOVA) was applied to each response variable, considering product type as the fixed factor. When a significant effect was detected (*p* < 0.05), means were compared using Tukey’s Honest Significant Difference (Tukey’s HSD) post-hoc test. Statistical significance was set at *p* < 0.05. Microbiological results were analysed descriptively only. Microbial counts were reported as CFU/g and interpreted in relation to the guideline values considered in the present study, while *Salmonella* spp. and *Listeria* *monocytogenes* were reported as absent or present in 25 g. Purchase intention data were also analysed descriptively only and expressed as the percentage of consumers selecting each response category for each product. Therefore, purchase intention responses were not submitted to ANOVA or other inferential statistical tests. Results are presented as mean ± standard deviation.

## 3. Results

### 3.1. Proximate Composition and Energy Content

Proximate composition of the developed fish products is depicted in Table 3. Due to different recipes requiring different ingredients, there is a wide variation in the analysed parameters, with significant differences across all parameters except ash content (*p* = 0.323), with an average value of 1.68%.

HBC showed the highest energy content (*p* < 0.05), with 176 kcal/100 g of product, whereas HHL had the highest total fibre content (*p* < 0.05), with 1.76 g/100 g of product. JFF presented significantly higher moisture and protein contents (*p* < 0.05), reaching 78.3 and 16.9 g/100 g of product, respectively. In contrast, HBC showed significantly higher fat and carbohydrate contents (*p* < 0.05), with values of 4.9 and 20.0 g/100 g of product, respectively.

### 3.2. Amino Acid Profile

The amino acid profile of the developed fish products is presented in Table 4. Significant differences (*p* < 0.05) were observed among the products for all identified amino acids, as well as for the total essential and non-essential amino acids, except for tyrosine and tryptophan, which did not differ significantly (*p* > 0.05; averaging 9.2 and 1.3 g/100 g of protein, respectively).

Lysine was the essential amino acid that contributed most to the total essential amino acid content in all products. Although a statistically significant difference was observed (*p* = 0.04), the total essential amino acid content was similar across most products, averaging 54.4 g/100 g of protein. Overall, the consumption of these fish products contributes approximately 18–20% towards meeting adult indispensable amino acid requirements.

When both essential and non-essential amino acids were considered, glutamic acid was the most abundant amino acid in HMF. Lysine content differed significantly among products (*p* < 0.05), with higher levels observed in HBC and PB (averaging 19.0 g/100 g of protein), corresponding to approximately 40–44% of the adult lysine requirement. The remaining products presented lower lysine levels, contributing between 15% and 34% to adult lysine requirements.

HMF showed the highest threonine content (6.7 g/100 g protein; *p* < 0.001), providing 29% of the adult threonine requirement. The remaining fish products had lower threonine content, contributing between 18% and 26% of the adult threonine requirement.

### 3.3. Vitamin Profile

The B-complex vitamins of the fish products are presented in Table 5. Significant differences (*p* < 0.001) were observed among developed fish products for all B-complex vitamins.

While HHL showed significantly higher contents of vitamins B1 and B9 (*p* < 0.05; 0.29 mg/100 g and 8.64 μg/100 g, respectively), corresponding to 24.2% and 3.5% of the adequate intake (AdInt) for vitamins B1 and B9, respectively, PB showed significantly lower contents (*p* < 0.05; 0.09 mg/100 g and 2.21 μg/100 g, respectively). The B1 content of PB, however, was not significantly different (*p* > 0.05) from that of BF. The remaining products displayed intermediate B1 and B9 contents, with significant (*p* < 0.05) differences between them, contributing 7.5–20.8% and 0.9–2.8% of the AdInt for vitamins B1 and B9, respectively. JFF and BF contained the highest concentrations of vitamins B2 and B3 (*p* < 0.05; 0.19 mg/100 g and 2.25 mg/100 g, respectively), contributing approximately 14% of the AdInt for both vitamins. Furthermore, HHL had the lowest level of B2 (0.05 mg/100 g), and PB had the lowest level of B3 (0.11 mg/100 g). The other products showed intermediate values, with significant differences (*p* < 0.05) between them. For vitamin B5, JFF, HHL, and HMF exhibited the highest contents (*p* < 0.05; averaging 1.88 mg/100 g; 37.8% of the AdInt), whereas PB contained the lowest (0.52 mg/100 g). JFF also had the highest B12 content (*p* < 0.05; 2.33 μg/100 g; 58.3% of the AdInt), while HHL had the lowest, though not significantly different from PB (*p* > 0.05; averaging 1.33 μg/100 g).

The tocopherol profile of the fish products is depicted in Table 6. δ-Tocopherol was only detected in PB, with a content of 0.004 mg/100 g and no significant differences were observed among fish products for β-tocopherol content (*p* = 0.052), which averaged 0.03 mg/100 g.

HBC showed the highest α-tocopherol content (*p* < 0.05; 1.83 mg/100 g), corresponding to 14.1% of the adequate intake for vitamin E, whereas PB presented the lowest content (0.40 mg/100 g; 3.1% of the AdInt). The remaining fish products exhibited intermediate levels, with significant differences among them (*p* < 0.05), and their contributions ranged from 6.4% to 10.4% of the AdInt for vitamin E.

For γ-tocopherol, HBC and HMF had the highest contents (*p* < 0.05; averaging 0.06 mg/100 g), which were not significantly different (*p* > 0.05) from HHL (0.05 mg/100 g). JFF contained the lowest content (0.02 mg/100 g), while PB showed an intermediate content (0.04 mg/100 g).

### 3.4. Fatty Acids Profile, Fatty Acid Ratios and Lipid Quality Indices

Regarding the fatty acids (FA) partial sums, PB showed the highest proportion of saturated fatty acids (SFA; *p* < 0.05; 31.18 g/100 g of total FA), whereas HMF presented a lipid profile dominated by monounsaturated fatty acids (MUFA; 67.86 g/100 g of total FA; Table 7). Furthermore, HBC contained the highest proportion of polyunsaturated fatty acids (PUFA; *p* < 0.05; 52.36 g/100 g of total FA), with the majority belonging to the n-6 PUFA family (51.38 g/100 g of total FA). Conversely, BF presented the highest n-3 PUFA content (14.67 g/100 g of total FA). The remaining products exhibited intermediate contents for the main FA groups, with significant (*p* < 0.05) differences between them.

For the FA ratios and lipid quality indices, BF showed the highest values of n-3 PUFA/n-6 PUFA, and PI (1.32 and 88.12, respectively), all of which were significantly higher (*p* < 0.05) than those of the other products. The highest P/S and h/H values were presented by HBC (4.49 and 10.56, respectively), whereas PB displayed the highest AI and TI values (0.43 and 0.72, respectively). The remaining products showed intermediate values for these ratios and indices, all of which differed significantly (*p* < 0.05) from one another.

The FA profiles of the developed fish products are quite complete, with 50 individual FA present across products. The FA profile of these products included 18 SFA (Table 8), 15 MUFA (Table 9), and 17 PUFA (Table 10). Of the six products analysed, complete quantification of all individual FA was achieved in HHL, JFF, and BF.

All individual SFAs differed significantly (*p* < 0.05) among the fish products, except C13:0, which showed no significant differences (*p* = 0.246; averaging 0.03 g/100 g of total FA).

The BF product showed significantly higher (*p* < 0.05) contents of C14:0 (2.07 g/100 g of total FA), aiC15:0 (0.38 g/100 g of total FA), C17:0 (1.23 g/100 g of total FA), C19:0 (0.22 g/100 g of total FA) and C21:0 (0.06 g/100 g of total FA) compared with the other products. However, the C19:0 content in BF did not differ significantly (*p* > 0.05) from that observed in PB and HHL. Furthermore, BF, together with HHL, presented the highest (*p* < 0.05) contents of iC15:0 and C15:0, averaging 0.80 g/100 g of total FA and 0.51 g/100 g of total FA, respectively.

Conversely, the PB product showed significantly higher (*p* < 0.05) contents of C10:0 (0.75 g/100 g of total FA), C12:0 (0.41 g/100 g of total FA), iC16:0 (0.19 g/100 g of total FA), C16:0 (26.0 g/100 g of total FA), and C20:0 (0.63 g/100 g of total FA) than the other products. Furthermore, PB, together with BF, presented significantly higher (*p* < 0.05) contents of iC17:0 (0.48 g/100 g of total FA), aiC17:0 (0.21 g/100 g of total FA), and C18:0 (4.46 g/100 g of total FA), although the latter did not differ significantly (*p* > 0.05) from the content observed in HHL.

Lastly, the HBC product showed the highest (*p* < 0.05) contents of C22:0 (0.60 g/100 g of total FA) and C24:0 (0.17 g/100 g of total FA), with the latter not differing significantly (*p* > 0.05) from the content presented by JFF.

The individual MUFA identified in this study are depicted in Table 9. All individual MUFA contents presented significant differences (*p* < 0.05) between products, except C16:1 *trans* (*p* = 0.081; averaging 0.55 g/100 g of total FA).

The BF product showed significantly higher (*p* < 0.05) contents of several MUFA, including C16:1 (*cis*-9) (8.42 g/100 g of total FA), C17:1 *cis*-7 (1.01 g/100 g of total FA), C18:1 *cis*-11 (5.36 g/100 g of total FA), C19:1 (0.17 g/100 g of total FA), and C20:1 *cis*-11 (3.00 g/100 g of total FA). However, the C19:1 content in BF did not differ significantly (*p* > 0.05) from that found in the HHL, PB, and HMF products.

In contrast, the PB product contained significantly higher (*p* < 0.05) contents of C14:1 (0.85 g/100 g of total FA), C16:1 (*cis*-7) (0.45 g/100 g of total FA), C16:1 *cis*-11 (1.30 g/100 g of total FA), and C22:1 *cis*-11 (0.11 g/100 g of total FA). The HHL product also demonstrated significantly higher (*p* < 0.05) contents of C17:1 *cis*-10 (0.64 g/100 g of total FA) and C20:1 *cis*-9 (1.84 g/100 g of total FA).

Additionally, HHL, together with PB, BF, exhibited significantly higher (*p* < 0.05) average contents of C15:1 (0.20 g/100 g of total FA) and C18:1 *cis*-6 (0.32 g/100 g of total FA). Among all products, HMF showed the highest (*p* < 0.05) content of C18:1 *cis*-9, reaching 60.80 g/100 g of total FA.

Lastly, the individual PUFA identified in this study are depicted in Table 10. All individual PUFA contents presented significant differences (*p* < 0.05) between products, except C18:2 (*trans*-9, *cis*-12) n-6 (*p* = 0.204; averaging 0.16 g/100 g of total FA).

The BF product showed significantly higher (*p* < 0.05) contents of most PUFA, including C18:2 (*trans*-9, *trans*-12) n-6 (0.30 g/100 g of total FA), C18:3 n-3 (4.00 g/100 g of total FA), γ-C18:3 n-6 (0.13 g/100 g of total FA), C20:2 n-6 (0.90 g/100 g of total FA), C20:3 n-3 (1.07 g/100 g of total FA), C20:4 n-6 (2.91 g/100 g of total FA), C20:4 n-3 (1.58 g/100 g of total FA), C20:5 n-3 (2.94 g/100 g of total FA), C22:5 n-3 (2.32 g/100 g of total FA), and C22:6 n-3 (3.14 g/100 g of total FA).

In addition, BF, together with JFF, presented significantly higher (*p* < 0.05) contents of C18:4 n-3, averaging 0.45 g/100 g of total FA. Similarly, BF and PB showed significantly higher (*p* < 0.05) contents of C20:3 n-6, averaging 0.38 g/100 g of total FA. PB also exhibited significantly higher (*p* < 0.05) contents of C22:3, although the difference was not significant (*p* > 0.05) when compared with BF, averaging 0.41 g/100 g of total FA.

Finally, HHL, along with PB and BF, showed significantly higher (*p* < 0.05) contents of C22:2 n-6, averaging 0.07 g/100 g of total FA. In contrast, HBC presented the highest (*p* < 0.05) content of C18:2 (*cis*-9, *cis*-12) n-6 (51.05 g/100 g of total FA), while HHL showed significantly higher (*p* < 0.05) contents of C22:4 n-6 (0.15 g/100 g of total FA).

### 3.5. Microbiological Evaluation

The highest microbial counts were observed in PB for all microbial indicator groups analysed, as observed in Table 11. Microorganisms at 30 °C ranged from 4.20 × 10^4^ CFU/g in HHL to 8.70 × 10^6^ CFU/g in PB, while microorganisms at 7 °C ranged from 3.10 × 10^4^ CFU/g in BF to 6.60 × 10^6^ CFU/g in PB. *Pseudomonas* spp. counts ranged from 2.50 × 10^4^ CFU/g in JFF to 1.50 × 10^6^ CFU/g in PB. *Enterobacteriaceae* counts ranged from 1.40 × 10^1^ CFU/g in HMF to 7.00 × 10^5^ CFU/g in PB. *Escherichia coli* was below the detection limit in all products, and *Salmonella* spp. and *Listeria monocytogenes* were absent.

**Table 11:** Microbiological evaluation of developed fish products.

	HHL	HMF	HBC	PB	BF	JFF
*n*	3	3	3	3	3	3
Count (CFU/g)						
Microorganisms at 30 °C	4.20 × 10^4^	4.10 × 10^5^	7.30 × 10^4^	8.70 × 10^6^	4.90 × 10^4^	5.50 × 10^4^
Microorganisms at 7 °C	6.10 × 10^4^	2.10 × 10^5^	3.90 × 10^4^	6.60 × 10^6^	3.10 × 10^4^	3.50 × 10^4^
*Pseudomonas* spp.	6.50 × 10^4^	5.40 × 10^5^	7.60 × 10^4^	1.50 × 10^6^	3.30 × 10^4^	2.50 × 10^4^
*Enterobacteriaceae*	2.90 × 10^2^	1.40 × 10^1^	1.70 × 10^3^	7.00 × 10^5^	5.00 × 10^2^	2.50 × 10^3^
*Escherichia coli*	<1	<1	<1	<1	<1	<1
Search (Absent—A; or present—P)						
*Salmonella* spp.	A/25 g	A/25 g	A/25 g	A/25 g	A/25 g	A/25 g
*Listeria monocytogenes*	A/25 g	A/25 g	A/25 g	A/25 g	A/25 g	A/25 g

HHL—Hamburger with herbs and lemon; HMF—Hamburger with Mediterranean flavour; HBC—Breaded Hamburger; PB—Powerbites; JFF—Japanese Flavoured Fillet; BF—Breaded Fillet; CFU/g—colony-forming units.

### 3.6. Texture Analysis

Ingredient-wise, the developed products were grouped into two groups: compound and whole fillet. The texture parameters of toughness and firmness are presented in Table 12. JFF, together with HBC and BF, exhibited significantly higher (*p* < 0.05) toughness and firmness values, averaging 10.97 N/mm·s and 2.47 N/mm, respectively, whereas the remaining fish products showed lower values for both parameters.

### 3.7. Sensory Analysis

The sensory scores for the six developed fish products, each with distinct compositions and flavour profiles, is depicted in Table 13.

BF was the most favourably perceived product, achieving the highest scores across all sensory attributes. JFF also showed strong sensory performance, ranking second for most parameters and presenting a balanced sensory profile, although it was slightly outperformed by HBC in appearance and colour.

The hamburger formulations showed a more moderate sensory performance. Among them, HBC was the best rated, with relatively high scores for appearance (4.00) and colour (3.98). However, its scores for the remaining parameters were lower than those of the fillet-based products, particularly for flavour and aftertaste. HHL and HMF presented similar and generally intermediate sensory scores, with overall acceptance values of 2.95 and 3.03, respectively. In contrast, PB consistently received the lowest scores across nearly all sensory attributes.

Purchase intention analysis (Table 14) provides further insight into how these sensory perceptions translate into potential market behaviour.

Consistent with the sensory results, BF shows the strongest purchase potential, with 29.5% of respondents indicating they would buy the product and an additional 45.5% responding “maybe yes,” resulting in a total positive purchase intention of 75.0%. JFF also demonstrates high commercial promise, with 11.6% definite buyers and 39.5% inclined buyers, amounting to 51.1% positive purchase intention. Although it shows a higher neutral response (34.9%) compared to BF, the low rejection rates (“maybe not” and “would not buy”) suggest that JFF is broadly acceptable.

Among the hamburger products, HBC again performs better than HHL and HMF, with 17.3% of respondents stating they would buy it and 38.5% indicating “maybe yes”, totalling 55.8%, a positive purchase tendency higher than JFF’s. In contrast, HHL and HMF show lower definite purchase intent (7.0% and 1.8%, respectively) and higher levels of hesitation or rejection, reflecting their more modest sensory performance. PB shows the weakest purchase intention tendency, without definitive purchase intentions, and only 10.6% expressing a positive inclination. High neutral (40.4%) and rejection responses (48.9% combined for “maybe not” and “would not buy”).

## 4. Discussion

### 4.1. Proximate Composition

The nutritional differences observed among the products appear to be mainly driven by their formulation, particularly the proportion of fish, the presence of breaded coatings, and the incorporation of fat- or fibre-rich ingredients. The higher energy, fat, and carbohydrate content observed in HBC may be explained by the contribution of breading ingredients and sunflower oil, together with its lower relative proportion of fish. In contrast, the higher moisture and protein contents observed in JFF were associated with its higher fish content, reflecting the naturally high water and protein composition of fish muscle. Products containing more fibre-rich ingredients, such as hamburgers and powerbites, showed increased fibre levels. Overall, these findings indicate that the nutritional profile of the developed fish products is strongly influenced not only by the amount of fish incorporated but also by the type and quantity of additional ingredients used, particularly breading ingredients, oils, and fibre sources.

### 4.2. Protein Quality and Amino Acid Composition

Proteins derived from fish are widely recognised for their high biological value, mainly due to their balanced essential amino acid (EAA) profile and high digestibility [51]. In this study, all developed fish products exhibited a complete profile of EAA, confirming the high nutritional quality of catfish protein even after processing. The high contribution of lysine observed in most products is consistent with previous reports on freshwater and marine fish, where lysine is often the predominant EAA and plays a key role in supporting tissue growth and maintenance [52]. This is particularly relevant from a nutritional standpoint, as lysine is frequently limiting in cereal-based diets [23]. The absence of significant differences in tryptophan content among products is also noteworthy. Such findings are in line with previous evidence indicating that tryptophan is relatively stable during conventional thermal processing, with marked losses occurring mainly under severe treatment conditions [53]. Similar observations have been reported in processed fish protein products, where amino acid profiles were largely preserved, and differences in tryptophan content were minimal across formulations [54].

Variations observed in certain amino acids, particularly lysine, among products can largely be attributed to technological treatments and formulation differences. Processing steps such as mincing, mixing, and the incorporation of non-fish ingredients may influence protein structure, functionality, and relative amino acid proportions [23,52]. Nevertheless, the overall amino acid profiles remained consistent with those of high-quality protein sources, reinforcing the suitability of *S. glanis* fillets and processing scraps for the development of protein-rich foods. This is in agreement with previous reports on restructured fish products, in which appropriate processing conditions were associated with the maintenance of protein quality and the preservation of EAA content [54,55].

### 4.3. Vitamin Profile

B-complex vitamins play essential roles in human metabolism, acting as coenzymes in energy production, amino acid metabolism, and neurological function. Fish is an important dietary source of several B-complex vitamins, particularly thiamine (B1), niacin (B3), pantothenic acid (B5), and cobalamin (B12) [27,29,30,32]. The presence of these vitamins in all developed fish products confirms the nutritional relevance of catfish as a micronutrient source.

Differences observed in B-vitamin contents among products can be explained by varying levels of fish incorporation and the specific ingredients used in each formulation. Products with higher proportions of fish muscle generally showed higher concentrations of vitamins B3 and B12, which is consistent with the literature indicating that these vitamins are predominantly associated with animal-derived tissues [56,57]. Conversely, dilution effects caused by cereal-based or vegetable ingredients likely contributed to lower vitamin concentrations in compound products, as also suggested by studies on cereal-based foods reformulated with fish ingredients, in which higher fish incorporation increased the micronutrient density of the final products [58].

Regarding vitamin E, α-tocopherol was the dominant tocopherol detected. The variability in tocopherol content among products can be attributed both to the intrinsic vitamin E levels of catfish and to formulation choices. Products containing sunflower oil exhibited higher α-tocopherol concentrations, which is expected given the well-documented richness of sunflower oil in vitamin E [59]. This highlights the potential of formulation strategies to enhance the micronutrient profile of fish-based products without compromising their sensory properties.

### 4.4. Fatty Acid Profile and Nutritional Implications

The lipid fraction of the developed products was characterised by a predominance of unsaturated FA, which is nutritionally advantageous and consistent with previous reports on freshwater catfish species [51]. Palmitic acid (C16:0) was the major SFA, a common feature in both freshwater and marine fish [51,60], while linoleic acid (C18:2 n-6) was the most abundant PUFA, particularly in products containing vegetable-derived ingredients.

HBC showed the highest P/S ratio, which was primarily associated with its higher C18:2 (*cis*-9, *cis*-12) n-6 content. By contrast, the lower P/S ratio in HMF appeared to reflect its lower contents of C18:2 (*cis*-9, *cis*-12) n-6 and C18:3 (9,12,15) n-3, whereas in PB this result was likely related to the higher proportions of C16:0 and C18:0. From a nutritional standpoint, the P/S ratio is commonly used as an indicator of the balance between PUFA and SFA, with higher values generally reflecting a more favourable lipid profile and potential relevance for cardiovascular health. A P/S ratio above 0.45 is generally considered desirable [35]. Therefore, the values observed for HMF and PB were below the recommended threshold, indicating a less favourable P/S balance in these products. These values are comparable to the lower range reported for 22 commercially important marine fish species, where P/S ratios varied from 0.26 to 0.79, and lower values were associated with reduced PUFA contents [61]. However, they were lower than those reported for canned fish products purchased on the Bulgarian market, for which the P/S ratio ranged from 0.58 to 1.26 [62]. Regarding the h/H index, HBC showed the most favourable value, suggesting a more favourable balance between hypocholesterolemic and hypercholesterolemic FA. Since higher h/H values are considered nutritionally more desirable [63], this finding agrees with the higher PUFA content observed in this product and suggests comparatively better lipid quality. Moreover, the h/H value obtained for HBC was consistent with the values reported for canned fish products, where all samples presented h/H ratios above 1.00 [62].

The balance between n-3 PUFA and n-6 PUFA is of particular nutritional relevance, as excessive n-6 PUFA intake relative to n-3 PUFA has been associated with increased cardiovascular risk [64]. The higher n-3 PUFA content, together with the lower n-6 PUFA content, in BF explains the lower and more favourable n-6 PUFA/n-3 PUFA ratio observed in this product. These characteristics also contributed to its higher PI value, as a greater degree of unsaturation is associated with an increased PI and, therefore, a higher susceptibility to oxidation. Despite these differences, the n-6 PUFA/n-3 PUFA ratio remained within the recommended threshold (≤4) [64] in all products, supporting their nutritional suitability in terms of FA balance.

The higher AI and TI values recorded for PB were likely associated with a greater contribution of SFA, especially C14:0 and C16:0. Overall, these characteristics indicate a less favourable nutritional lipid profile and suggest a higher atherogenic and thrombogenic potential [63].

Taken together, these results show that formulation markedly influenced the lipid quality of the developed products. Although all formulations met recommended n-6/n-3 values and contained predominantly unsaturated FA, HBC emerged as the nutritionally most favourable product, whereas PB showed the least favourable lipid-related health indices. BF, while nutritionally advantageous in terms of n-3 PUFA content, may require greater attention regarding oxidative stability.

### 4.5. Microbiological Quality and Food Safety Considerations

Fish is a food that is very susceptible to contamination by microorganisms and therefore has a short shelf life and high health risk, especially when good hygiene and conservation practices are not implemented throughout the food chain [65]. Since there is no European regulation for products including *S. glanis*, the guideline values defined by INSA Guidelines (2019) were used, specifically the group 2D as a reference, which includes fish dishes such as sushi or tartare [66].

According to ICMSF [65] and the INSA Guidelines [66] and the guideline value of 1 × 10^6^ CFU/g for aerobic mesophiles in fish, only PB exceeded the recommended limit. This result indicates a comparatively higher microbial load in this product, suggesting the need to improve hygiene and process control.

*Enterobacteriaceae* counts ranged from 1.40 × 10^1^ to 7.00 × 10^5^ CFU/g, with the highest value also observed in PB. Considering the INSA guideline value of less than 1 × 10^5^ CFU/g [66], this result suggests the need to improve hygiene and process control, particularly the hygienic conditions of the raw material, the handling of products during their production and the possibility of the presence of mesophilic pathogenic microorganisms [65].

Overall, the microbiological results indicate that, although the tested pathogens were absent at the time of analysis, the elevated aerobic mesophilic counts observed in PB and the *Enterobacteriaceae* levels detected in some products highlight the need for improved hygiene and process control before commercial application.

### 4.6. Texture, Sensory Acceptance, and Purchase Intention

Texture is a critical determinant of consumer perception and acceptance of fish-based products, particularly when comparing intact fillet products with restructured or compound formulations. Overall, fillet-based products exhibited higher firmness and toughness values than compound products. This trend may be related to the preservation of muscle fibre structure and the higher proportion of intact myofibrillar proteins in fillet-based products. In contrast, compound formulations are more affected by mincing, ingredient dispersion, and the incorporation of binders or non-fish ingredients, which may disrupt the continuity of the muscle matrix and contribute to softer textures. Similar trends have been reported for fish products in which intact muscle structure resulted in firmer textures, while minced or restructured formulations tend to present softer and less cohesive textures due to protein fragmentation and ingredient dispersion [67].

These instrumental textural differences were consistent with the sensory results. BF and the JFF achieved higher scores for texture, juiciness, flavour, and overall acceptance, suggesting that preservation of a recognised fillet structure contributed positively to consumer evaluation. In the case of BF, the breaded coating may also have contributed to sensory acceptance by improving surface texture and providing sensory attributes commonly associated with breaded fish products. Previous studies have shown that battering and breading systems can influence coating crispness, hardness, moisture retention, fat uptake, and consumer acceptability in fish and seafood products [15,16,68]. In contrast, PB, the compound product incorporating the highest proportion of plant-based ingredients, showed lower sensory scores and weaker purchase intention. Although this formulation was designed to increase nutritional diversity and sustainability, its lower fish content, softer texture, and more heterogeneous ingredient matrix may have contributed to its lower sensory acceptance.

A coherent relationship between sensory acceptance and purchase intention was observed across all products. Products receiving higher hedonic scores consistently showed higher positive purchase intention, supporting the relevance of sensory liking as a key driver of consumer acceptance of fish and seafood products. Previous studies have identified sensory quality, convenience, familiarity, and product format as important factors influencing fish and seafood consumption and purchasing behaviour [9,69,70]. Therefore, the higher acceptance of breaded and flavoured fillet products may be related to their familiar format and favourable sensory performance. Such formats may facilitate the introduction of products developed from less familiar or underutilised freshwater fish species by aligning product characteristics with consumer expectations. However, broader conclusions regarding perceived risk or consumer confidence would require dedicated consumer studies specifically designed to evaluate these aspects. These findings reinforce that the successful valorisation of invasive fish species depends not only on nutritional quality and sustainability, but also on product format, texture, sensory quality, and consumer acceptability.

### 4.7. Implications for the Valorisation of an Invasive Species

Overall, the integration of nutritional, technological, microbiological, and sensory data demonstrates that *S. glanis* can be transformed into value-added food products with significant potential for human consumption. From an industrial standpoint, the development of fillet-based and restructured products may enhance the technological versatility of this species, enabling its incorporation into different product categories and market segments. Moreover, the use of both fillets and processing by-products aligns with circular economy principles by promoting more efficient raw material utilisation, minimising waste, and adding value to fractions that are commonly underused. In this context, the valorisation of *S. glanis* may support invasive species management while promoting more sustainable fish-based products. However, market acceptance will depend on meeting consumer expectations for texture, flavour, convenience, and familiarity. Familiar formats, such as breaded or flavoured fillets, may ease market introduction, whereas innovative composite products may require further optimisation.

### 4.8. Limitations of the Study

Although this study provides a preliminary characterisation of the developed *S. glanis* products and supports their qualitative potential for valorisation within a circular economy framework, two important limitations should be acknowledged. First, shelf-life assessment was not conducted, which is particularly relevant since the products are intended for refrigerated storage. Future research should therefore evaluate microbiological stability, sensory changes, lipid oxidation, and nutritional quality during refrigerated storage, in order to define suitable storage conditions and estimate shelf-life. Second, quantitative sustainability and economic feasibility indicators were not assessed. Future studies should include parameters such as processing yield, percentage of edible biomass recovered, waste reduction, energy and water use, carbon footprint, and life-cycle assessment analysis. Together, this data will be essential to determine the environmental and economic viability of scaling up *S. glanis*-based products and to support future industrial applications and commercial validation.

## 5. Conclusions

*Silurus glanis* is an invasive species of the Tagus River that must be actively controlled to prevent irreversible damage to the aquatic ecosystem. This study demonstrated that *S. glanis* can be successfully valorised through the development of diversified food products while preserving its nutritional quality and achieving differentiated levels of consumer acceptance by integrating fillets and fish scraps into value-added formulations.

The development of value-added foods from *S. glanis* represents a viable strategy to simultaneously promote sustainable protein consumption, valorise processing by-products and contribute to invasive species management. All developed products exhibited high-quality protein, characterised by a complete essential amino acid profile. Additionally, the added ingredients contributed meaningful amounts of B-complex vitamins and vitamin E, depending on formulations.

The lipid fraction of the products was dominated by unsaturated fatty acids, with favourable fatty acid ratios and health-related lipid indices, especially in fillet-based products. These characteristics reinforce the nutritional suitability of *S. glanis* as a lean freshwater fish with potential benefits for cardiovascular health. Variations in fatty acid composition and micronutrient content among products further illustrate the strong influence of processing and formulation strategies on nutritional outcomes.

From a consumer perspective, it was shown that by mixing with different ingredients and flavours, it’s possible to create products appealing to the public, with potential economic value.

## Figures and Tables

**Table 1:** Ingredient composition of the developed fish products. Ingredients are expressed as a percentage of the final formulation.

Ingredient/Ingredient Group	Technological Function	HHL (%)	HMF (%)	HBC (%)	PB (%)	JFF (%)	BF (%)
*Silurus glanis*	Main protein source	82.6	85.1	52.0	50.0	90.0	66.0
Rice flour	Binder/structuring agent	4.5	4.5	4.3	5.0	–	–
Pea fibre	Water-binding/texture improvement	3.2	2.5	2.9	3.0	–	–
Vegetable fibre	Water-binding/texture improvement	1.5	1.5	1.5	1.5	–	–
Methylcellulose	Binder/structuring agent	–	–	0.2	–	0.1	–
Sunflower oil	Juiciness/mouthfeel	1.0	–	6.0	–	3.2	–
Olive oil	Juiciness/mouthfeel	–	2.0	–	–	–	–
Lemon and garlic marinade	Flavouring/seasoning	3.0	–	13.5	3.4	–	16.4
Teriyaki sauce	Flavouring/seasoning	–	–	–	–	3.6	–
Lemon juice	Flavouring/acidity	1.5	–	–	–	–	–
Garlic/powdered garlic	Flavouring	–	1.0	0.3	2.0	–	–
Herbs, spices and seasoning blends ^1^	Flavouring/product differentiation	0.6	1.1	0.3	–	–	–
Vegetables ^2^	Flavour/texture/product differentiation	–	–	–	33.0	–	–
Breading ingredients ^3^	Coating/texture contrast	–	–	17.0	–	–	16.0
Preservative	Preservation	1.0	1.0	1.0	1.0	1.0	1.0
Salt	Seasoning/technological function	0.8	0.8	1.0	0.6	–	0.7
Maltodextrin	Carrier/texture support	0.5	0.5	–	0.5	–	–
Water	Moisture adjustment	–	–	–	–	2.1	–
Caramel colouring	Colour adjustment	–	–	0.1	–	–	–

HHL—hamburger with herbs and lemon; HMF—hamburger with Mediterranean flavour; HBC—breaded hamburger; PB—powerbites; JFF—Japanese flavoured fillet; BF—breaded fillet. ^1^ Herbs, spices, and seasoning blends include basil, parsley, Italian seasoning blend, powdered tomato, paprika, white pepper, and caramel colouring, depending on the formulation; ^2^ Vegetables include corn, purple onion, carrot, and pea; ^3^ Breading ingredients include breadcrumbs, corn flakes, and corn flour.

**Table 2:** Adequate intake (AdInt) values for vitamins (expressed as mg/day).

Vitamin	Adequate Intake	Reference
E ^1^	13	[26]
B1	1.2	[27]
B2 ^2^	1.3	[28]
B3 ^2^	16.2	[29]
B5	5	[30]
B9 ^3^	250	[31]
B12 ^3^	4	[32]

^1^ as α-tocopherol; ^2^ Considered the average requirement value; ^3^ expressed as µg/day.

**Table 3:** Energy content (expressed as kcal/100 g of fish product) and proximate composition (expressed as g/100 of fish product) of developed fish products.

	HHL	HMF	HBC	PB	JFF	BF	*p*
*n*	3	3	3	3	3	3	
Energy	108.13 ± 4.17 ^bc^	113.56 ± 1.49 ^b^	176.29 ± 8.48 ^a^	98.84 ± 3.15 ^cd^	86.24 ± 2.18 ^d^	121.84 ± 8.10 ^b^	<0.001
Moisture	71.94 ± 0.65 ^b^	71.69 ± 0.49 ^b^	59.7 ± 1.16 ^d^	73.82 ± 0.54 ^b^	78.27 ± 0.53 ^a^	68.15 ± 1.41 ^c^	<0.001
Protein	14.72 ± 0.80 ^ab^	15.06 ± 0.66 ^ab^	12.53 ± 1.9 ^c^	10.05 ± 0.51 ^c^	16.89 ± 0.26 ^a^	14.83 ± 0.78 ^ab^	<0.001
Fat	1.38 ± 0.27 ^bc^	2.09 ± 0.47 ^b^	4.94 ± 0.37 ^a^	0.74 ± 0.17 ^cd^	1.51 ± 0.09 ^bc^	0.51 ± 0.02 ^d^	<0.001
Carbohydrate	8.34 ± 1.30 ^c^	7.97 ± 0.86 ^c^	19.66 ± 3.06 ^a^	12.15 ± 0.08 ^bc^	0.95 ± 0.70 ^d^	13.83 ± 2.86 ^b^	<0.001
Total fibre	1.76 ± 0.22 ^a^	1.31 ± 0.23 ^ab^	1.51 ± 0.56 ^ab^	1.67 ± 0.37 ^ab^	0.64 ± 0.44 ^b^	1.31 ± 0.33 ^ab^	0.040
Ash	1.86 ± 0.13	1.88 ± 0.02	1.65 ± 0.43	1.57 ± 0.18	1.74 ± 0.21	1.37 ± 0.49	0.323

Values are expressed as mean ± Standard Deviation (SD); Different letters in a given row indicate significant differences at *p* < 0.05 (Tukey’s HSD test); HHL—Hamburger with herbs and lemon; HMF—Hamburger with Mediterranean flavour; HBC—Breaded Hamburger; PB—Powerbites; JFF—Japanese Flavoured Fillet; BF—Breaded Fillet.

**Table 4:** Amino acid profile of developed fish products (expressed as g/100 g of protein).

	HHL	HMF	HBC	PB	JFF	BF	*p*
*n*	3	3	3	3	3	3	
Aspartic acid/Asparagine	8.59 ± 0.56 ^a^	9.09 ± 0.56 ^a^	8.50 ± 0.56 ^a^	8.13 ± 0.56 ^a^	5.89 ± 0.56 ^b^	7.67 ± 0.56 ^a^	0.020
Glutamic acid/Glutamine	14.29 ± 0.77 ^a^	11.10 ± 0.77 ^bc^	15.38 ± 0.77 ^a^	13.44 ± 0.77 ^ab^	7.51 ± 0.77 ^d^	9.37 ± 0.77 ^cd^	<0.001
Serine	0.90 ± 0.14 ^c^	4.92 ± 0.14 ^a^	3.97 ± 0.14 ^b^	4.03 ± 0.14 ^b^	4.16 ± 0.14 ^b^	4.19 ± 0.14 ^b^	<0.001
Histidine	4.90 ± 0.20 ^a^	4.70 ± 0.20 ^a^	3.56 ± 0.20 ^cd^	4.03 ± 0.20 ^bc^	3.30 ± 0.20 ^d^	4.52 ± 0.20 ^ab^	0.001
Threonine	4.80 ± 0.19 ^cd^	6.71 ± 0.19 ^a^	4.21 ± 0.19 ^e^	4.46 ± 0.19 ^de^	5.98 ± 0.19 ^b^	5.27 ± 0.19 ^cd^	<0.001
Glycine	4.20 ± 0.12 ^b^	5.02 ± 0.12 ^a^	3.32 ± 0.12 ^d^	3.75 ± 0.12 ^c^	4.03 ± 0.12 ^bc^	4.28 ± 0.12 ^b^	<0.001
Arginine	7.19 ± 0.24 ^a^	5.97 ± 0.24 ^b^	4.70 ± 0.24 ^c^	5.09 ± 0.24 ^c^	7.29 ± 0.24 ^a^	5.13 ± 0.24 ^cd^	<0.001
Alanine	3.70 ± 0.25 ^b^	5.76 ± 0.25 ^a^	3.48 ± 0.25 ^b^	3.96 ± 0.25 ^b^	5.30 ± 0.25 ^a^	5.04 ± 0.25 ^a^	<0.001
Tyrosine	11.39 ± 1.63	8.99 ± 1.63	6.96 ± 1.63	8.91 ± 1.63	10.23 ± 1.63	8.80 ± 1.63	0.585
Methionine	2.70 ± 0.20 ^b^	3.01 ± 0.20 ^ab^	2.02 ± 0.20 ^c^	1.77 ± 0.20 ^c^	3.62 ± 0.20 ^a^	3.30 ± 0.20 ^a^	<0.001
Valine	5.89 ± 0.42 ^b^	8.67 ± 0.42 ^a^	6.72 ± 0.42 ^b^	6.72 ± 0.42 ^b^	8.15 ± 0.42 ^a^	9.04 ± 0.42 ^a^	0.001
Tryptophan	1.00 ± 0.96	0.95 ± 0.96	3.16 ± 0.96	1.20 ± 0.96	0.91 ± 0.96	0.89 ± 0.96	0.569
Phenylalanine	6.29 ± 0.26 ^bc^	6.50 ± 0.26 ^b^	5.67 ± 0.26 ^cd^	5.16 ± 0.26 ^d^	6.56 ± 0.26 ^ab^	7.30 ± 0.26 ^a^	0.001
Isoleucine	3.00 ± 0.13 ^c^	5.07 ± 0.13 ^a^	4.94 ± 0.13 ^a^	3.96 ± 0.13 ^b^	4.98 ± 0.13 ^a^	5.27 ± 0.13 ^a^	<0.001
Leucine	5.89 ± 0.21 ^b^	6.66 ± 0.21 ^a^	5.43 ± 0.21 ^b^	5.45 ± 0.21 ^b^	6.79 ± 0.21 ^a^	6.83 ± 0.21 ^a^	0.001
Lysine	15.38 ± 1.30 ^bc^	6.82 ± 1.30 ^d^	18.06 ± 1.30 ^ab^	20.01 ± 1.30 ^a^	15.39 ± 1.30 ^bc^	13.04 ± 1.30 ^cd^	<0.001
Essentials AA	49.85 ± 1.09 ^bc^	49.15 ± 1.09 ^c^	53.77 ± 1.09 ^a^	52.69 ± 1.09 ^ab^	55.64 ± 1.09 ^a^	55.51 ± 1.09 ^a^	0.004
Nonessentials AA	50.15 ± 1.09 ^ab^	50.85 ± 1.09 ^a^	46.23 ± 1.09 ^c^	47.31 ± 1.09 ^bc^	44.36 ± 1.09 ^c^	44.49 ± 1.09 ^c^	0.004

Values are expressed as mean ± Standard Deviation (SD); Different letters in a given row indicate significant differences at *p* < 0.05 (Tukey’s HSD test); HHL—Hamburger with herbs and lemon; HMF—Hamburger with Mediterranean flavour; HBC—Breaded Hamburger; PB—Powerbites; JFF—Japanese Flavoured Fillet; BF—Breaded Fillet.

**Table 5:** B-Complex vitamin content of developed fish products (expressed as mg/100 g of fish product).

	HHL	HMF	HBC	PB	JFF	BF	*p*
*n*	3	3	3	3	3	3	
B1	0.29 ± 0.01 ^a^	0.25 ± 0.01 ^b^	0.21 ± 0.01 ^b^	0.09 ± 0.01 ^d^	0.15 ± 0.01 ^c^	0.11 ± 0.01 ^cd^	<0.001
B2	0.05 ± 0.01 ^d^	0.14 ± 0.01 ^b^	0.11 ± 0.01 ^c^	n.d.	0.19 ± 0.01 ^a^	0.18 ± 0.01 ^a^	<0.001
B3	1.07 ± 0.05 ^c^	1.38 ± 0.05 ^b^	1.17 ± 0.05 ^c^	0.11 ± 0.05 ^d^	2.28 ± 0.05 ^a^	2.22 ± 0.05 ^a^	<0.001
B5	1.92 ± 0.10 ^a^	1.87 ± 0.10 ^a^	1.43 ± 0.10 ^b^	0.52 ± 0.10 ^c^	1.86 ± 0.10 ^a^	1.43 ± 0.10 ^b^	<0.001
B9 *	8.64 ± 0.20 ^a^	6.40 ± 0.20 ^c^	6.30 ± 0.20 ^c^	2.21 ± 0.20 ^e^	7.08 ± 0.20 ^b^	5.62 ± 0.20 ^d^	<0.001
B12 *	1.30 ± 0.02 ^e^	1.40 ± 0.02 ^d^	1.96 ± 0.02 ^b^	1.35 ± 0.02 ^de^	2.33 ± 0.02 ^a^	1.62 ± 0.02 ^c^	<0.001

* Expressed as µg/100 g of fish product; Values are expressed as mean ± Standard Deviation (SD); Different letters in a given row indicate significant differences at *p* < 0.05 (Tukey’s HSD test); HHL—Hamburger with herbs and lemon; HMF—Hamburger with Mediterranean flavour; HBC—Breaded Hamburger; PB—Powerbites; JFF—Japanese Flavoured Fillet; BF—Breaded Fillet; n.d., not detected, i.e., below the limit of detection (0.0457 mg/100 mL).

**Table 6:** Tocopherol profile of developed fish products (expressed as mg/100 g of fish product).

	HHL	HMF	HBC	PB	JFF	BF	*p*
*n*	3	3	3	3	3	3	
α-tocopherol	1.12 ± 0.04 ^c^	0.83 ± 0.04 ^d^	1.83 ± 0.04 ^a^	0.40 ± 0.04 ^e^	1.35 ± 0.04 ^b^	1.07 ± 0.04 ^c^	<0.001
β-tocopherol	0.04 ± 0.01	0.02 ± 0.01	0.06 ± 0.01	0.01 ± 0.01	0.03 ± 0.01	0.04 ± 0.01	0.052
γ-tocopherol	0.05 ± 0.004 ^ab^	0.06 ± 0.004 ^a^	0.05 ± 0.004 ^a^	0.04 ± 0.004 ^b^	0.02 ± 0.004 ^c^	0.03 ± 0.004 ^c^	<0.001
δ-tocopherol	n.d.	n.d.	n.d.	0.004 ± 0.0002	n.d.	n.d.	-

Values are expressed as mean ± Standard Deviation (SD); Different letters in a given row indicate significant differences at *p* < 0.05 (Tukey’s HSD test); HHL—Hamburger with herbs and lemon; HMF—Hamburger with Mediterranean flavour; HBC—Breaded Hamburger; PB—Powerbites; JFF—Japanese Flavoured Fillet; BF—Breaded Fillet; n.d., not detected, i.e., below the limit of detection (0.0013 mg/100 mL).

**Table 7:** Fatty acid (FA) partial sums (expressed as g of FA/100 g of total FA), FA ratios, and lipid quality indices of developed fish products.

	HHL	HMF	HBC	PB	JFF	BF	*p*
*n*	3	3	3	3	3	3	
SFA	22.75 ± 1.35 ^bc^	20.99 ± 1.35 ^cd^	12.60 ± 1.46 ^e^	31.18 ± 1.46 ^a^	17.44 ± 1.35 ^d^	25.72 ± 1.26 ^b^	<0.001
MUFA	45.01 ± 2.26 ^b^	67.86 ± 2.26 ^a^	34.46 ± 2.45 ^c^	48.81 ± 2.45 ^b^	42.07 ± 2.26 ^b^	44.55 ± 2.112 ^b^	<0.001
PUFA	29.15 ± 3.571 ^bc^	9.67 ± 3.571 ^e^	52.36 ± 3.857 ^a^	17.34 ± 3.857 ^de^	38.90 ± 3.571 ^b^	25.43 ± 3.340 ^cd^	<0.001
*Trans*	0.73 ± 0.13 ^ab^	0.42 ± 0.13 ^bc^	0.17 ± 0.14 ^c^	0.51 ± 0.14 ^bc^	0.47 ± 0.13 ^bc^	0.92 ± 0.13 ^a^	<0.001
n-6 PUFA	24.07 ± 3.05 ^c^	6.66 ± 3.05 ^d^	51.38 ± 3.29 ^a^	13.30 ± 3.29 ^d^	34.77 ± 3.05 ^b^	11.18 ± 2.85 ^d^	<0.001
n-3 PUFA	5.44 ± 1.365 ^b^	3.65 ± 1.365 ^bc^	1.13 ± 1.475 ^c^	4.17 ± 1.475 ^bc^	4.60 ± 1.365 ^b^^c^	14.67 ± 1.277 ^a^	<0.001
P/S	1.55 ± 0.14 ^c^	0.38 ± 0.10 ^d^	4.49 ± 0.10 ^a^	0.15 ± 0.14 ^d^	2.71 ± 0.10 ^b^	0.65 ± 0.10 ^d^	<0.001
n-3 PUFA/n-6 PUFA	0.35 ± 0.12 ^bc^	0.56 ± 0.12 ^b^	0.02 ± 0.13 ^c^	0.23 ± 0.13 ^bc^	0.18 ± 0.12 ^c^	1.32 ± 0.12 ^a^	<0.001
h/H	4.36 ± 0.38 ^c^	4.13 ± 0.38 ^c^	10.56 ± 0.41 ^a^	2.45 ± 0.41 ^d^	6.50 ± 0.38 ^b^	2.89 ± 0.36 ^d^	<0.001
AI	0.23 ± 0.03 ^c^	0.23 ± 0.03 ^c^	0.10 ± 0.04 ^d^	0.43 ± 0.04 ^a^	0.17 ± 0.03 ^cd^	0.33 ± 0.03 ^b^	<0.001
TI	0.39 ± 0.06 ^b^	0.40 ± 0.06 ^b^	0.25 ± 0.07 ^b^	0.72 ± 0.07 ^a^	0.30 ± 0.06 ^b^	0.33 ± 0.06 ^b^	<0.001
PI	53.15 ± 8.83 ^b^	25.77 ± 8.83 ^c^	57.79 ± 9.53 ^b^	34.70 ± 9.53 ^bc^	59.30 ± 8.83 ^b^	88.12 ± 8.26 ^a^	<0.001

Values are expressed as mean ± Standard Deviation (SD); Different letters in a given row indicate significant differences at *p* < 0.05 (Tukey’s HSD test); HHL—Hamburger with herbs and lemon; HMF—Hamburger with Mediterranean flavour; HBC—Breaded Hamburger; PB—Powerbites; JFF—Japanese Flavoured Fillet; BF—Breaded Fillet.

**Table 8:** Saturated fatty acids (SFA; expressed as g of FA/100 g of total FA) of developed fish products.

	HHL	HMF	HBC	PB	JFF	BF	*p*
*n*	3	3	3	3	3	3	
C10:0	0.10 ± 0.06 ^b^	n.d.	n.d.	0.75 ± 0.06 ^a^	0.03 ± 0.06 ^b^	0.08 ± 0.06 ^b^	<0.001
C12:0	0.10 ± 0.06 ^b^	0.02 ± 0.08 ^b^	n.d.	0.41 ± 0.07 ^a^	0.06 ± 0.07 ^b^	0.13 ± 0.06 ^b^	0.006
C13:0	0.03 ± 0.01	n.d.	n.d.	n.d.	0.02 ± 0.02	0.05 ± 0.01	0.246
C14:0	1.18 ± 0.12 ^b^	0.39 ± 0.12 ^cd^	0.20 ± 0.11 ^d^	1.36 ± 0.12 ^b^	0.57 ± 0.12 ^c^	2.07 ± 0.12 ^a^	<0.001
iC15:0	0.75 ± 0.05 ^a^	0.19 ± 0.04 ^c^	0.06 ± 0.05 ^c^	n.d.	0.31 ± 0.04 ^b^	0.84 ± 0.04 ^a^	<0.001
aiC15:0	0.16 ± 0.03 ^c^	0.08 ± 0.03 ^d^	0.03 ± 0.02 ^d^	0.32 ± 0.02 ^b^	0.16 ± 0.02 ^c^	0.38 ± 0.02 ^a^	<0.001
C15:0	0.51 ± 0.02 ^a^	0.14 ± 0.02 ^d^	0.05 ± 0.02 ^e^	0.42 ± 0.02 ^b^	0.22 ± 0.02 ^c^	0.51 ± 0.02 ^a^	<0.001
iC16:0	0.07 ± 0.04 ^b^	n.d.	n.d.	0.19 ± 0.03 ^a^	0.004 ± 0.06 ^b^	0.03 ± 0.03 ^b^	0.024
C16:0	11.34 ± 0.63 ^c^	16.67 ± 0.56 ^b^	7.74 ± 0.56 ^d^	26.0 ± 0.73 ^a^	10.1 ± 0.52 ^c^	15.12 ± 0.56 ^b^	<0.001
iC17:0	0.29 ± 0.04 ^b^	0.09 ± 0.03 ^c^	0.04 ± 0.05 ^c^	0.48 ± 0.05 ^a^	0.12 ± 0.07 ^bc^	0.48 ± 0.05 ^a^	<0.001
aiC17:0	0.12 ± 0.01 ^b^	0.06 ± 0.01 ^c^	0.03 ± 0.01 ^c^	0.21 ± 0.01 ^a^	0.02 ± 0.02 ^c^	0.21 ± 0.01 ^a^	<0.001
C17:0	0.80 ± 0.10 ^b^	0.38 ± 0.09 ^c^	0.18 ± 0.13 ^c^	0.80 ± 0.13 ^b^	0.49 ± 0.19 ^bc^	1.23 ± 0.13 ^a^	0.002
C18:0	4.14 ± 0.17 ^ab^	2.47 ± 0.14 ^d^	3.38 ± 0.14 ^c^	4.54 ± 0.15 ^a^	3.91 ± 0.15 ^b^	4.38 ± 0.14 ^a^	<0.001
C19:0	0.15 ± 0.03 ^ab^	0.06 ± 0.03 ^b^	n.d.	0.16 ± 0.04 ^ab^	0.07 ± 0.04 ^b^	0.22 ± 0.03 ^a^	0.012
C20:0	0.27 ± 0.03 ^cd^	0.32 ± 0.03 ^c^	0.23 ± 0.03 ^d^	0.63 ± 0.03 ^a^	0.24 ± 0.03 ^cd^	0.44 ± 0.03 ^b^	<0.001
C21:0	0.03 ± 0.01 ^b^	0.03 ± 0.003 ^b^	n.d.	n.d.	0.02 ± 0.01 ^b^	0.06 ± 0.05 ^a^	0.006
C22:0	0.39 ± 0.03 ^c^	0.12 ± 0.03 ^e^	0.60 ± 0.03 ^a^	0.21 ± 0.03 ^d^	0.51 ± 0.03 ^b^	0.13 ± 0.03 ^de^	<0.001
C24:0	0.09 ± 0.02 ^b^	0.05 ± 0.01 ^b^	0.17 ± 0.01 ^a^	n.d.	0.19 ± 0.01 ^a^	0.07 ± 0.02 ^b^	<0.001

Values are expressed as mean ± Standard Deviation (SD); Different letters in a given row indicate significant differences at *p* < 0.05 (Tukey’s HSD test); HHL—Hamburger with herbs and lemon; HMF—Hamburger with Mediterranean flavour; HBC—Breaded Hamburger; PB—Powerbites; JFF—Japanese Flavoured Fillet; BF—Breaded Fillet; n.d., not detected, i.e., below the limit of detection.

**Table 9:** Monounsaturated fatty acids (MUFA; expressed as g of FA/100 g of total FA) of developed fish products.

	HHL	HMF	HBC	PB	JFF	BF	*p*
*n*	3	3	3	3	3	3	
C14:1	0.28 ± 0.09 ^bc^	0.02 ± 0.16 ^cd^	0.07 ± 0.16 ^cd^	0.85 ± 0.09 ^a^	0.09 ± 0.09 ^c^	0.38 ± 0.09 ^b^	<0.001
C15:1	0.19 ± 0.03 ^a^	n.d.	0.03 ± 0.04 ^b^	0.20 ± 0.03 ^a^	0.08 ± 0.03 ^b^	0.19 ± 0.03 ^a^	<0.001
C16:1 *cis*-7	0.18 ± 0.02 ^c^	0.08 ± 0.02 ^d^	0.03 ± 0.02 ^d^	0.45 ± 0.02 ^a^	0.09 ± 0.02 ^d^	0.29 ± 0.02 ^b^	<0.001
C16:1 *cis*-9	5.39 ± 0.34 ^b^	3.63 ± 0.27 ^c^	0.68 ± 0.30 ^d^	5.97 ± 0.34 ^b^	3.74 ± 0.30 ^c^	8.42 ± 0.34 ^a^	<0.001
C16:1 *trans*	0.72 ± 0.15 ^ab^	0.31 ± 0.13 ^b^	0.19 ± 0.21 ^c^	0.83 ± 0.21 ^ab^	0.30 ± 0.29 ^bc^	0.92 ± 0.21 ^a^	0.081
C16:1 *cis*-11	0.90 ± 0.04 ^c^	0.27 ± 0.03 ^e^	0.06 ± 0.04 ^f^	1.30 ± 0.04 ^a^	0.39 ± 0.03 ^d^	1.06 ± 0.03 ^b^	<0.001
C17:1 *cis*-7	0.68 ± 0.04 ^b^	0.49 ± 0.03 ^c^	0.14 ± 0.04 ^d^	0.63 ± 0.03 ^b^	0.47 ± 0.03 ^c^	1.01 ± 0.03 ^a^	<0.001
C17:1 *cis*-10	0.64 ± 0.12 ^a^	0.04 ± 0.14 ^c^	n.d.	0.13 ± 0.24 ^bc^	0.06 ± 0.11 ^c^	0.28 ± 0.09 ^b^	0.018
C18:1 *cis*-6	0.24 ± 0.05 ^ab^	0.34 ± 0.05 ^a^	0.04 ± 0.09 ^b^	0.33 ± 0.05 ^a^	0.12 ± 0.05 ^b^	0.35 ± 0.05 ^a^	<0.001
C18:1 *cis*-9	29.98 ± 1.46 ^b^	60.80 ± 1.32 ^a^	32.53 ± 2.09 ^b^	27.95 ± 2.09 ^bc^	29.95 ± 2.95 ^bc^	23.11 ± 2.09 ^c^	<0.001
C18:1 *cis*-11	4.18 ± 0.37 ^b^	3.41 ± 0.48 ^b^	1.04 ± 0.97 ^c^	3.43 ± 0.39 ^b^	1.68 ± 0.48 ^c^	5.36 ± 0.34 ^a^	<0.001
C19:1	0.13 ± 0.02 ^ab^	0.10 ± 0.03 ^ab^	n.d.	0.12 ± 0.03 ^ab^	0.07 ± 0.02 ^b^	0.17 ± 0.03 ^a^	<0.001
C20:1 *cis*-9	1.84 ± 0.13 ^a^	0.11 ± 0.13 ^c^	0.05 ± 0.15 ^c^	0.39 ± 0.13 ^bc^	0.13 ± 0.13 ^c^	0.55 ± 0.15 ^b^	<0.001
C20:1 *cis*-11	0.39 ± 0.21 ^c^	0.64 ± 0.18 ^c^	0.35 ± 0.29 ^c^	1.61 ± 0.29 ^b^	0.73 ± 0.41 ^bc^	3.00 ± 0.29 ^a^	<0.001
C22:1 *cis*-11	0.08 ± 0.01 ^b^	0.02 ± 0.01 ^d^	n.d.	0.11 ± 0.01 ^a^	0.06 ± 0.01 ^c^	0.09 ± 0.01 ^b^	<0.001

Values are expressed as mean ± Standard Deviation (SD); Different letters in a given row indicate significant differences at *p* < 0.05 (Tukey’s HSD test); HHL—Hamburger with herbs and lemon; HMF—Hamburger with Mediterranean flavour; HBC—Breaded Hamburger; PB—Powerbites; JFF—Japanese Flavoured Fillet; BF—Breaded Fillet; n.d., not detected, i.e., below the limit of detection.

**Table 10:** Polyunsaturated fatty acids (PUFA; expressed as g of FA/100 g of total FA) of developed fish products.

	HHL	HMF	HBC	PB	JFF	BF	*p*
*n*	3	3	3	3	3	3	
C18:2 (*trans*-9, *trans*-12) n-6	0.20 ± 0.03 ^b^	0.07 ± 0.03 ^c^	n.d.	0.13 ± 0.04 ^bc^	0.11 ± 0.04 ^c^	0.30 ± 0.03 ^a^	<0.001
C18:2 (*trans*-9, *cis*-12) n-6	0.15 ± 0.03	0.10 ± 0.03	0.20 ± 0.04	0.11 ± 0.04	0.20 ± 0.06	0.20 ± 0.04	0.204
C18:2 (*cis*-9, *cis*-12) n-6	16.96 ± 0.41 ^d^	5.85 ± 0.27 ^f^	51.05 ± 0.32 ^a^	19.03 ± 0.41 ^c^	34.83 ± 0.36 ^b^	8.85 ± 0.32 ^e^	0.001
C18:3 n-3	1.56 ± 0.17 ^c^	1.56 ± 0.15 ^c^	0.49 ± 0.24 ^d^	3.07 ± 0.24 ^b^	1.88 ± 0.34 ^c^	4.00 ± 0.24 ^a^	<0.001
γ-C18:3 n-6	0.05 ± 0.01 ^b^	0.05 ± 0.01 ^b^	-	0.08 ± 0.02 ^b^	0.04 ± 0.01 ^b^	0.13 ± 0.01 ^a^	<0.001
C18:4 n-3	0.11 ± 0.02 ^c^	0.09 ± 0.02 ^c^	0.09 ± 0.02 ^c^	0.30 ± 0.02 ^b^	0.49 ± 0.03 ^a^	0.40 ± 0.02 ^a^	<0.001
C20:2 n-6	0.30 ± 0.07 ^bc^	0.14 ± 0.06 ^c^	0.06 ± 0.10 ^c^	0.48 ± 0.10 ^b^	0.18 ± 0.14 ^bc^	0.90 ± 0.10 ^a^	0.001
C20:3 n-6	0.13 ± 0.03 ^b^	0.09 ± 0.02 ^b^	0.04 ± 0.04 ^b^	0.33 ± 0.04 ^a^	0.13 ± 0.05 ^b^	0.42 ± 0.04 ^a^	<0.001
C20:3 n-3	0.42 ± 0.02 ^c^	0.14 ± 0.02 ^e^	0.03 ± 0.03 ^f^	0.53 ± 0.03 ^b^	0.28 ± 0.02 ^d^	1.07 ± 0.03 ^a^	<0.001
C20:4 n-6	1.01 ± 0.20 ^bc^	0.51 ± 0.18 ^c^	0.24 ± 0.28 ^c^	1.67 ± 0.28 ^b^	0.82 ± 0.40 ^bc^	2.91 ± 0.28 ^a^	<0.001
C20:4 n-3	0.22 ± 0.03 ^c^	0.17 ± 0.03 ^c^	0.05 ± 0.03 ^d^	0.61 ± 0.05 ^b^	0.59 ± 0.04 ^b^	1.58 ± 0.03 ^a^	<0.001
C20:5 n-3	0.81 ± 0.17 ^cd^	0.59 ± 0.15 ^d^	0.28 ± 0.24 ^d^	1.74 ± 0.24 ^b^	1.45 ± 0.33 ^bc^	2.94 ± 0.24 ^a^	<0.001
C22:2 n-6	0.05 ± 0.01 ^a^	n.d.	n.d.	0.08 ± 0.01 ^a^	0.03 ± 0.1 ^b^	0.08 ± 0.01 ^a^	<0.001
C22:3	0.27 ± 0.04 ^bc^	0.12 ± 0.05 ^de^	0.04 ± 0.05 ^e^	0.45 ± 0.04 ^a^	0.18 ± 0.04 ^cd^	0.37 ± 0.04 ^ab^	<0.001
C22:4 n-6	0.15 ± 0.001 ^a^	n.d.	n.d.	n.d.	0.01 ± 0.001 ^b^	0.02 ± 0.001 ^b^	0.012
C22:5 n-3	0.62 ± 0.17 ^c^	0.39 ± 0.15 ^c^	0.19 ± 0.24 ^c^	1.41 ± 0.24 ^b^	0.96 ± 0.34 ^bc^	2.32 ± 0.24 ^a^	0.001
C22:6 n-3	0.98 ± 0.28 ^bc^	0.61 ± 0.25 ^c^	0.28 ± 0.39 ^c^	1.82 ± 0.39 ^b^	1.38 ± 0.55 ^bc^	3.14 ± 0.39 ^a^	0.003

Values are expressed as mean ± Standard Deviation (SD); Different letters in a given row indicate significant differences at *p* < 0.05 (*Tukey’s HSD* test); HHL—Hamburger with herbs and lemon; HMF—Hamburger with Mediterranean flavour; HBC—Breaded Hamburger; PB—Powerbites; JFF—Japanese Flavoured Fillet; BF—Breaded Fillet; n.d., not detected, i.e., below the limit of detection.

**Table 12:** Toughness (expressed as N/mm·s) and firmness (expressed as N/mm) texture parameters for the developed fish products.

	HHL	HMF	HBC	PB	JFF	BF	*p*
*n*	3	3	3	3	3	3	
Toughness	5.64 ± 1.14 ^b^	5.32 ± 1.42 ^b^	9.38 ± 0.93 ^ab^	6.70 ± 0.93 ^b^	13.24 ± 1.14 ^a^	10.30 ± 1.14 ^ab^	0.006
Firmness	1.27 ± 0.22 ^bc^	0.88 ± 0.22 ^c^	2.43 ± 0.18 ^a^	0.77 ± 0.18 ^c^	2.24 ± 0.22 ^ab^	2.74 ± 0.22 ^a^	<0.001

Values are expressed as mean ± Standard Deviation (SD); Different letters in a given row indicate significant differences at *p* < 0.05 (Tukey’s HSD test); HHL—Hamburger with herbs and lemon; HMF—Hamburger with Mediterranean flavour; HBC—Breaded Hamburger; PB—Powerbites; JFF—Japanese Flavoured Fillet; BF—Breaded Fillet.

**Table 13:** Sensory evaluation scores for the developed fish products, from the 5-point hedonic scale used (1—dislike very much; 5—like very much).

	HHL	HMF	HBC	PB	JFF	BF	*p*
*n*	3	3	3	3	3	3	
Appearance	2.89 ± 0.94 ^c^	3.36 ± 0.93 ^bc^	4.00 ± 0.91 ^a^	3.02 ± 1.01 ^bc^	3.54 ± 0.95 ^ab^	4.02 ± 0.82 ^a^	<0.001
Colour	2.88 ± 0.89 ^c^	3.41 ± 0.84 ^b^	3.98 ± 0.84 ^a^	3.29 ± 0.94 ^bc^	3.54 ± 0.89 ^ab^	4.02 ± 0.76 ^a^	<0.001
Texture	2.75 ± 0.97 ^c^	3.05 ± 0.94 ^bc^	3.40 ± 1.14 ^ab^	2.58 ± 0.87 ^c^	3.50 ± 0.97 ^ab^	3.93 ± 0.88 ^a^	<0.001
Juiciness	3.32 ± 0.91 ^abc^	3.18 ± 1.07 ^bc^	3.40 ± 0.96 ^abc^	2.92 ± 1.00 ^c^	3.58 ± 0.78 ^ab^	3.74 ± 1.00 ^a^	0.002
Flavour	3.13 ± 1.03 ^bc^	2.90 ± 1.05 ^c^	3.23 ± 1.10 ^abc^	2.83 ± 0.95 ^c^	3.52 ± 0.81 ^ab^	3.80 ± 1.00 ^a^	<0.001
Smell	2.95 ± 0.86 ^b^	3.00 ± 0.90 ^b^	3.25 ± 0.96 ^ab^	2.94 ± 0.88 ^b^	3.40 ± 0.93 ^ab^	3.64 ± 0.97 ^a^	0.001
Aftertaste	3.00 ± 0.91 ^ab^	2.79 ± 0.95 ^b^	3.04 ± 0.97 ^ab^	2.67 ± 0.94 ^b^	3.24 ± 0.89 ^a^	3.50 ± 1.05 ^a^	<0.001
Overall acceptance	2.95 ± 0.91 ^b^	3.03 ± 0.97^bc^	3.33 ± 1.00 ^bc^	2.86 ± 0.94 ^b^	3.47 ± 0.84 ^a^^b^	3.93 ± 0.87 ^a^	<0.001

Values are expressed as mean ± Standard Deviation (SD); Different letters in a given row indicate significant differences at *p* < 0.05 (Tukey’s HSD test); HHL—Hamburger with herbs and lemon; HMF—Hamburger with Mediterranean flavour; uHBC—Breaded Hamburger; PB—Powerbites; JFF—Japanese Flavoured Fillet; BF—Breaded Fillet.

**Table 14:** Purchase intentions (expressed as %) by developed fish products of tasters, using a 5-point scale (1—certainly would not buy; 5—certainly would buy).

	HHL	HMF	HBC	PB	JFF	BF
*n*	3	3	3	3	3	3
Certainly would buy	7.0	1.8	17.3	0.0	11.6	29.5
Maybe yes	31.6	22.8	38.5	10.6	39.5	45.5
Neutral	22.8	35.1	21.2	40.4	34.9	15.9
Maybe not	21.1	26.3	11.5	25.5	9.3	4.5
Certainly would not buy	17.5	14.0	11.5	23.4	4.7	4.5

HHL—Hamburger with herbs and lemon; HMF—Hamburger with Mediterranean flavour; HBC—Breaded Hamburger; PB—Powerbites; JFF—Japanese Flavoured Fillet; BF—Breaded Fillet. Purchase intention is expressed as the percentage of consumers selecting each response category. No inferential statistical analysis was performed for this variable.

## Data Availability

The original contributions presented in this study are included in the article. Further inquiries can be directed to the corresponding author.

## Abbreviations

The following abbreviations are used in this manuscript:

*S. glanis*	*Silurus glanis*
FA	Fatty acid(s)
HHL	Hamburger with herbs and lemon
HMF	Hamburger with mediterranean flavour
HBC	Breaded Hamburger
PB	Powerbites
JFF	Japanese flavoured fillet
BF	Breaded fillet
rPET	Recycled Polyethylene Terephthalate
PET	Polyethylene Terephthalate
AOAC	Association of Official Analytical Chemists
ISO	International Organization for Standardization
EU	European Union
HPLC	High-performance liquid chromatography
AdInt	Adequate intake
EFSA NDA	Panel on Nutrition, Novel Foods and Food Allergens of the European Food Safety Authority
FAME	Fatty acid methyl esters
AI	Atherogenicity index
TI	Thrombogenicity index
PI	Peroxidability index
h/H	Hypocholesterolemic/hypercholesterolemic ratio
GDPR	General Data Protection Regulation
SD	Standard deviation
EAA	Essential amino acid
NEAA	Nonessential amino acid
SFA	Saturated fatty acid
MUFA	Monounsaturated fatty acid
PUFA	Polyunsaturated fatty acid
CFU	Colony forming units
INSA	Instituto Nacional de Saúde Doutor Ricardo Jorge
ICMSF	International Commission on Microbiological Specifications for Foods
